# Effects of Dietary Nucleotide Supplementation on Performance, Profitability, and Disease Resistance of *Litopenaeus vannamei* Cultured in Indonesia under Intensive Outdoor Pond Conditions

**DOI:** 10.3390/ani12162036

**Published:** 2022-08-10

**Authors:** Romi Novriadi, Oriol Roigé, Sergi Segarra

**Affiliations:** 1 Aquaculture Department, Jakarta Technical University of Fisheries, Politeknik Ahli Usaha Perikanan, Ministry of Marine Affairs and Fisheries, Jl. Raya Pasar Minggu, Jati Padang, Jakarta 12520, Indonesia; 2 R&D Bioiberica S.A.U., Av. Dels Països Catalans 34, 08950 Esplugues de Llobregat, Spain

**Keywords:** nucleotides, crustacean aquaculture, nutrition, Pacific white shrimp, growth performance, profitability, disease resistance, *Vibrio harveyi*

## Abstract

**Simple Summary:**

In Indonesia, there is an increasing tendency to use vegetable protein sources as a replacement for fish meal in diets for Pacific white shrimp (PWS), *Litopenaeus vannamei*. This; however, involves a negative impact on shrimp health given the high content of anti-nutritional factors in such protein sources. As dietary nucleotides modulate the immune response, they might be able to counteract this effect by enhancing PWS immunity and improving their performance and resistance to diseases. In this study, we evaluated the effects of nucleotide supplementation in PWS receiving diets in which fish meal had been partially replaced with soybean meal. Our data show that dietary nucleotide supplementation leads to improved growth performance and profitability, as well as to higher resistance to *Vibrio harveyi*. These positive results indicate that dietary nucleotides could constitute a useful tool in the production of shrimp cultured under intensive open-pond systems.

**Abstract:**

This study evaluated the effects of dietary nucleotide supplementation in Pacific white shrimp, *Litopenaeus vannamei*, cultured in Indonesia. A total of 22,500 shrimp receiving diets in which fish meal (FM) had been partially replaced with vegetable protein sources were classified into five study groups (4500 shrimp/group) and received different diets for 110 days: 10FM (control group; 10% FM), 6FM (6% FM—low FM and no nucleotide supplementation), 10FMN (10% FM; 0.1% nucleotides), 8FMN (8% FM; 0.1% nucleotides) and 6FMN (6% FM; 0.1% nucleotides). Growth performance, body composition, total hemocyte count (THC), lysozyme activity, and hepatopancreas histopathology were assessed. Organoleptic evaluation and profitability assessments were also performed. In addition, shrimp resistance to a *Vibrio harveyi* challenge was studied in shrimps after having received the diets for 30 days. Results showed that reducing FM had a negative impact on growth performance and hepatopancreas morphology. Adding nucleotides resulted in better performance and profitability, a healthier histomorphological appearance of the hepatopancreas, and significantly higher survival rates upon challenge with *V. harveyi*, while it did not negatively affect organoleptic parameters. In conclusion, nucleotide supplementation could be useful for optimizing performance, profitability, and disease resistance in shrimp cultured under intensive outdoor pond conditions.

## 1. Introduction

Pacific white shrimp (PWS), *Litopenaeus vannamei* (Boone, 1931) is one of the most widely cultured shrimp species globally. They are produced not only in their native region in the Pacific ocean [1,2], but also in Southeast Asia [3,4]. The development and growth of the PWS industry can be explained by several factors, including their high demand [5,6,7], acceptance of formulated feeds [8,9,10], and the feasibility of culturing PWS intensively [7,11]. However, the rapid expansion of this species has also prompted an increased incidence of disease as well as a degradation of the physical environment, which can affect PWS growth and health conditions [12,13].

Over the past number of years, awareness has arisen around the concept that the most sustainable strategy for enhancing PWS growth performance and survival should rely on the development of functional diets that allow health and economic benefits beyond basic nutrition [14,15]. There are several functional products available that could contribute to such aims, including growth promoters, probiotics, prebiotics, immunomodulators, phytogenic substances, and organic acids, targeting intestinal health, stress, and disease resistance of aquatic organisms [15,16,17]. Nucleotides are immunomodulatory compounds, and they are the building blocks of DNA and RNA and are important for many physiological processes in living organisms. In PWS, the use of nucleotides has been described, as they can be supplied through the feed, and lead to enhanced disease resistance [18] and growth performance during the culture period [19]. Dietary nucleotide supplementation has also been proven to improve biological functions and to provide several health benefits in other animal species, including modulation of immunity, resistance to infection and enhancement of growth performance. More specifically, several publications support the benefits of Nucleoforce^®^, a proprietary brand of nucleotide-rich yeast extract developed by Bioiberica S.A.U. (Palafolls, Spain), which is highly sustainable, as it can be obtained through a fermentation process following a circular bioeconomy approach [20]. In a recent study with this nucleotide extract in PWS, our research group demonstrated a positive effect in PWS receiving diets in which fish meal (FM) had been partially replaced with soybean meal (SBM). In that study, although an improvement in performance parameters was observed, this effect did not reach the level of statistical significance [21]. One of the limitations of the study was that the effects of nucleotides were not evaluated up until the shrimp had reached their commercial consumption size.

In penaeid shrimp, *Vibrio harveyi* is recognized as a serious cause of disease, contributing to mass mortality during the grow-out production system [22,23,24,25,26]. From a biological perspective, supplementing diets with nucleotides seems especially adequate in the early stages, since rapid weight gain occurs during the juvenile stage [27]. To develop sustainable and economically adequate practical diets for PWS, and taking into account that in the coming years a reduction in the use of FM is expected, nucleotides could be used in shrimp feed formulations to counteract the negative effects of using SBM. Therefore, the aim of the present study was to evaluate the long-term effects of nucleotide supplementation on the performance, profitability, immune response, and resistance to *V. harveyi* of PWS fed a diet in which FM had been partially replaced with SBM and cultured under intensive outdoor pond conditions in Indonesia.

## 2. Materials and Methods

### 2.1. Experimental Diets

Diets were prepared with different FM inclusion levels and formulated with SBM, corn gluten meal, and wheat products as the main protein ingredients. The control diet (10FM, containing 10% FM) was designed to serve as a representative for commonly used diets for PWS production in the Indonesian market, as opposed to 6FM (6% FM), which represented a diet in which FM had been partially replaced with vegetable sources. Nucleotides (N, Nucleoforce^®^, Bioiberica, S.A.U., Palafolls, Spain) were supplemented at 0.1% (Table 1) in diets containing different FM levels: 10% (10FMN), 8% (8FMN) and 6% (6FMN). All diets were produced at the Karawang Aquaculture Development Center and manufactured using commercial methods with a twin-screw extruder (CXE 65 E, Jinan Shengrun, Jinan, China).

Prior to production, all ingredients were mixed in a paddle mixer (Marion Mixers, Inc., Marion, IA, USA) for a 100 kg batch, followed by grinding to a particle size of <200 µm using a disk mill (Jinan Shengrun, Jinan, China). The cooking-extrusion diets were exposed to an average of 110 °C for approximately 14 s in five-barrel sections, and the last section was maintained at 62 °C. Pressure at the die head was approximately 50 bars, and screw speed was maintained at 423 rpm. A portion of the feeds was extruded through 1 and 2 mm dies to produce 1.5 and 2.5 mm particles. Diets were dried in a pulse bed dryer (Jinan Shengrun, China) until moisture readings were below 6%. Pellets were dried at approximately 107 °C with an upper limit outflow air temperature of approximately 88 °C. Diets were then cooled at ambient air temperatures to achieve final moisture levels of less than 10%. All finished diets were bagged and stored in a temperature-controlled room until further use. Proximate and amino acid profile of the diets were analyzed at Saraswanti Indo Genetech Laboratory, Bogor, West Java, Indonesia and summarized in Table 2.

### 2.2. Growth Trial

The growth trial was conducted in two commercial ponds with a size of 20 × 30 m per pond at the Jakarta Technical University of Fisheries (Jakarta, Indonesia). A total of 22,500 PWS were obtained from Salira teknik Benur (Serang, Banten, Indonesia) and acclimatized to the culture system for one week in the nursery tank. Shrimp (1.06 ± 0.01 g initial mean weight) were then randomly distributed into 50 nets (450 shrimp per net) with a size of 2 × 2 × 1 m. Ten replicate groups of shrimp were administered different types of experimental diets (4500 shrimp per study group) using a nutrition research standard protocol for 110 days and fed by hand four times daily, at 07:00, 11:00, 15:00 and 20:00 h. Based on our historic results, feed inputs were pre-programmed assuming the normal growth of shrimp and feed conversion ratio of 1.5. Daily allowances of feed were adjusted based on the observed feed consumption, weekly shrimp counts, and mortality [21]. Uneaten feed, feces, and molts were removed by siphoning the aquaria prior to the first feeding.

### 2.3. Body Composition Analysis

Twenty shrimp from each study group were randomly sampled at the end of the trial and stored at −80 °C for body composition analysis. Prior to the proximate, energy, and amino acid analyses, dried whole shrimp were rigorously blended and chopped in a mixer according to the methods described by Helrich et al. [28]. All parameters were analyzed at the Saraswanti Indo Genetech Laboratory (Bogor, West Java, Indonesia).

### 2.4. Water Quality and Growth Sampling

Water pH, dissolved oxygen (DO), temperature, and salinity were measured four times daily using the Aqua TROLL 500 Multiparameter Sonde instrument and connected to the AquaEasy apps (Bosch, Singapore) for the data monitoring and recording system. Total ammonia nitrogen (TAN), nitrate, total general bacteria, and total *Vibrio* spp. were measured once a week, using standard protocols [29], at the Main Center for Brackishwater Aquaculture Development (Jepara, West Java, Indonesia) and Aquatic Animal Diagnostic Laboratory (Central Proteina Prima, Tangerang, Banten, Indonesia). At the end of the feeding period, all shrimp were grouped and individually weighed to calculate the final biomass, final body weight (FBW), percentage weight gain (PWG), feed conversion ratio (FCR), survival rate (SR), and thermal growth coefficient (TGC) as follows:

$$\mathrm{PWG}=\frac{\left(\mathrm{average}\;\mathrm{individual}\;\mathrm{final}\;\mathrm{weight}-\mathrm{average}\;\mathrm{individual}\;\mathrm{initial}\;\mathrm{weight}\right)}{\left(\mathrm{average}\;\mathrm{individual}\;\mathrm{initial}\;\mathrm{weight}\right)}\times 100$$

$$\mathrm{FCR}=\frac{\mathrm{feed}\;\mathrm{given}\;\left(g\right)}{\mathrm{live}\;\mathrm{weight}\;\mathrm{gain}\;\left(g\right)}$$

$$\mathrm{SR}=\frac{\mathrm{final}\;\mathrm{number}\;\mathrm{of}\;\mathrm{shrimp}}{\mathrm{initial}\;\mathrm{number}\;\mathrm{of}\;\mathrm{shrimp}}\times 100$$

$$\mathrm{TGC}=\frac{{\mathrm{FBW}}^{1/3}-{\;\mathrm{IBW}}^{1/3}}{\sum^{\;}\mathrm{TD}}\text{×}100$$

FBW (final body weight), IBW (initial body weight), T (water temperature in °C), and D (number of trial days) [21].

### 2.5. Total Hemocyte Count

**Figure 1:**
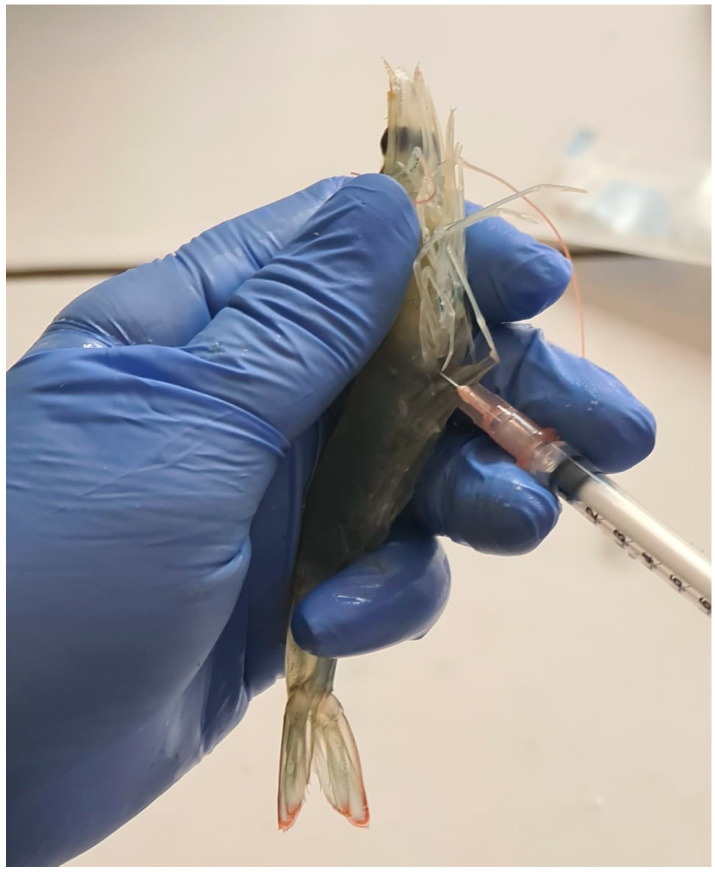
Withdrawal of hemolymph performed on one of the Pacific white shrimp (PWS) from the study. Hemolymph was obtained from the pleopod base of the second abdominal segment by using a sterile 1-mL syringe and a 25 G × 13 mm needle.

At the end of the growth trial, the hemolymph was sampled (Figure 1) from 50 shrimp of each study group, and the total hemocyte count (THC) and lysozyme activity were determined. Hemolymph (100 μL) of individual shrimp was withdrawn from the pleopod base of the second abdominal segment with a sterile 1 mL syringe (25 G × 13 mm needle). Before hemolymph extraction, the syringe was loaded with a precooled (4 °C) solution (10% EDTA Na2) used as an anticoagulant. The hemolymph with the anti-coagulant solution were diluted in 150 μL of formaldehyde (4%) and then 20 μL was placed on a hemocytometer (Neubauer) to determine the THC using an optical microscope (Olympus, DP72) following methods described by [30].

### 2.6. Lysozyme Activity Analysis

Lysozyme activity was measured using a lysozyme detection kit (Sigma-Aldrich, Cat. no. LY0100) according to the manufacturer’s instructions. The results of the lysozyme activity were defined by the lysis of *Micrococcus lysodeikticus* cells. The reactions were conducted at 25 °C, and the absorbance at 450 nm was measured on an ultraviolet-visible spectrophotometer (Perkin Elmer, Lambda XLS, Waltham, MA, USA).

$$\mathrm{Lysozyme}\;\mathrm{activity}\;\left(\frac{\mathrm{Units}}{\mathrm{mL}}\right)=\frac{(\Delta A450/\min\mathrm{Test}-\Delta A450/\min\mathrm{Blank})\;\left(\mathrm{df}\right)}{\left(0.001\right)\left(0.03\right)}$$

df = dilution factor; 0.001 = ΔA_450_ as per unit definition; 0.03 = volume (in mL) of enzyme solution [31].

### 2.7. Histology

Shrimp samples for the histopathological studies were selected randomly. Sections of approximately 0.5 cm was immediately preserved in Davidson’s at room temperature and then transferred to 70% ethanol solution (VWR, Radnor, PA, USA) until processed by standard histological analysis procedures [32]. Samples were dehydrated through a standard ethanol series to 100%, embedded in paraffin wax, and sectioned at 4 µm intervals for staining with hematoxylin-eosin (H&E) stain (Merck, Darmstadt, Germany). Images were acquired using a digital imaging microscope (Olympus BX41, Olympus Optical Co., Ltd., Tokyo, Japan).

### 2.8. Challenge Test

Samples of *Vibrio harveyi* were isolated from infected shrimp in East Java, from an area in which there is a high mortality due to this microorganism. Strain was then confirmed by using RT-PCR. Shrimp that had received the experimental diets for 30 days were used in a *Vibrio harveyi* challenge test, with 1 × 10^5^ CFU mL^−1^ administered intramuscularly. Shrimp mortality was observed every day for the 5-day challenge test, and the cumulative mortality rate was calculated.

### 2.9. Organoleptic Analysis

Organoleptic evaluations were carried out at the Jakarta shrimp research center (Jakarta, Indonesia). Firstly, bags of shrimp were inspected to detect any damage and stored at −34 °C in a freezer. Twenty-four hours before the organoleptic evaluation, shrimp were thawed at 0–4 °C in a refrigerator. The shrimp were then inspected for general appearance and color criteria according to Quality Index Method [33], and weighed individually using a digital scale. Individuals from the same treatment and tank were then cooked, based on laboratory preliminary tests carried out to establish the cooking time. Afterwards, they were collected, cooled for approximately 1 min in a bowl of icy water, and manually peeled, including the abdomen tissue.

The organoleptic evaluation method followed the guidelines for standard cooked shrimp. The shrimp were maintained at room temperature for 30 min before being distributed on the trays. Three shrimp were placed in the center of a dish and panelists received four packs of shrimp at a time. For the evaluation, each panelist rated the color, smell, texture, and taste using a nine-point hedonic scale, as well as commenting freely on their perceptions. All assessments took place in booths that complied with the general standards for the design of test rooms intended for sensory analyses. Lighting was daylight type light, at an average intensity of 780 lux. Panelists were recruited from PT. Sinergi Samudera Biru (Jakarta, Indonesia) and could be considered as an “internal trained panel” as they regularly consumed marine products and were already experienced in organoleptic testing. For the estimations, a double-blinded design with a scale of 1 to 5 was used, where a score of 5 was considered the most favorable. Panelists provided an independent observation on shrimp color, aroma, taste, and texture.

### 2.10. Profitability Analysis

The comparative cost–benefit evaluation regarding the use of the experimental feed was based on 1 production cycle using a 0.1 ha pond with a density of 200 PL/m^2^. The farm gate prices were based on January 2022 wholesale prices in Indonesia using Indonesian rupiah (IDR) as currency. The growth parameters in this study and the necessary investments were the main factors used to calculate the comparative profitability of each different diet. Gross margins were compared to calculate the profitability of each different study group.

### 2.11. Statistical Analysis

Growth parameters, total hemocyte counts, lysozyme activity, and the challenge test were analyzed using a regression and one-way analysis of variance (ANOVA) to determine significant differences among treatments, followed by Tukey’s multiple comparison tests to determine the difference between treatment means among the treatments. Score data on shrimp hepatopancreas histomorphological condition and organoleptic scores were treated as categorical data, tested for normality and homoscedasticity, and subsequently analyzed using a linear regression model. All statistical analyses were conducted using the SAS system (V9.4. SAS Institute, Cary, NC, USA).

## 3. Results

### 3.1. Water Quality

No remarkable alterations were noted in water quality parameters evaluated during the course of the trial.

### 3.2. Growth Performance

Growth performance and survival of PWS during the feeding trial are summarized in Table 3. In this study, shrimp receiving 10FM showed significantly higher FBW, PWG, TGC and average daily growth (ADG), and lower FCR, compared to 6FM. On the other hand, a positive impact was observed on performance following nucleotide supplementation, with 6FMN achieving significantly better results vs. 6FM in FBW, PWG, FCR, TGC and ADG. Numerically, better outcomes were also observed with 10FMN, compared to 10FM, but this positive impact of dietary nucleotide supplementation in shrimp receiving regular FM inclusion levels (10%) did not reach statistical significance.

### 3.3. Body Composition Analysis and Organoleptic Evaluation

Shrimp whole-body composition at the end of the growth trial is presented in Table 4. A higher whole-body protein content was measured in shrimp fed with nucleotides compared to non-supplemented groups, and a higher moisture content was observed in shrimp fed 6FM, compared to the rest of the groups.

### 3.4. Total Hemocyte Counts and Lysozyme Activity

**Figure 2:**
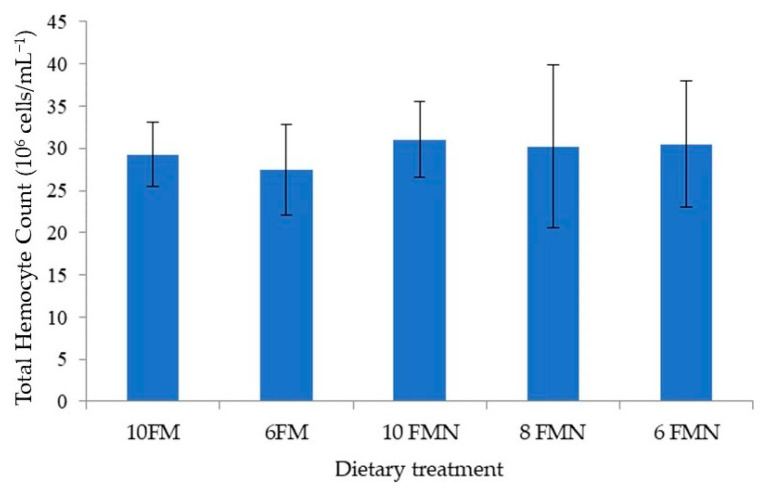
Pacific white shrimp (PWS) total hemocyte count (10^6^ cells/mL^−1^) at the end of the 110-day growth trial. 10FM (control group; 10% FM), 6FM (6% FM and no nucleotide supplementation), 10FMN (10% FM; 0.1% nucleotides), 8FMN (8% FM; 0.1% nucleotides) and 6FMN (6% FM; 0.1% nucleotides). Values represent the mean of ten replicates.

**Figure 3:**
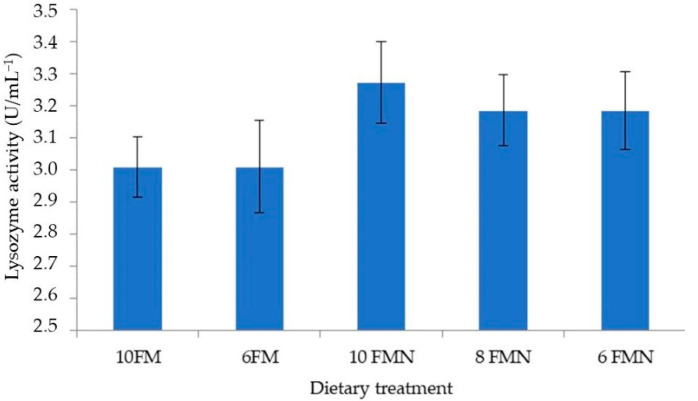
Pacific white shrimp (PWS) lysozyme activity (U/mL^−1^) at the end of the 110-day growth trial. 10FM (control group; 10% FM), 6FM (6% FM and no nucleotide supplementation), 10FMN (10% FM; 0.1% nucleotides), 8FMN (8% FM; 0.1% nucleotides) and 6FMN (6% FM; 0.1% nucleotides). Values represent the mean of ten replicates.

No statistically significant differences were found in THC (Figure 2) or lysozyme activity (Figure 3) between the different study groups, although numerically higher values were obtained with nucleotide supplementation.

### 3.5. Histomorphological Condition of Shrimp Hepatopancreas

**Figure 4:**
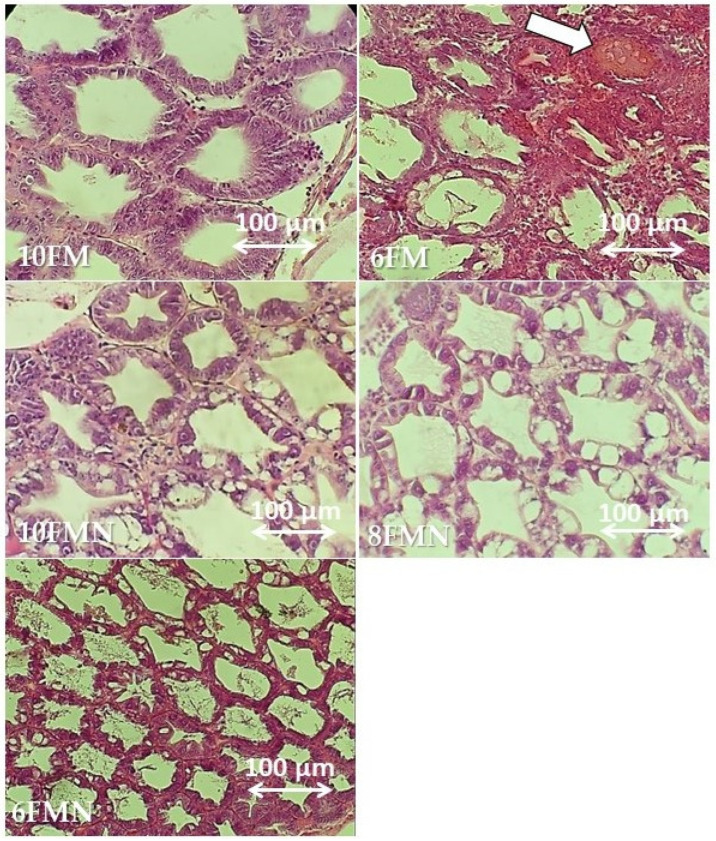
Representative histopathological images of hematoxylin and eosin-stained sections of the hepatopancreas from Pacific white shrimp (PWS) belonging to different study groups. 10FM (control group; 10% FM), 6FM (6% FM and no nucleotide supplementation), 10FMN (10% FM; 0.1% nucleotides), 8FMN (8% FM; 0.1% nucleotides) and 6FMN (6% FM; 0.1% nucleotides). The white arrow points to vacuolation observed in the 6FM group as a result of replacing fish meal with vegetable protein sources.

The effects of the different dietary treatments on hepatopancreas histology are depicted in Figure 4. FM10 featured a normal appearance, with the absence of vacuoles and intercellular spaces, and the intestinal epithelium showing an organized and normal position. Meanwhile, 6FM showed increased hemocyte infiltration, vacuolation, and enlarged nucleus compared to 10FM. The inclusion of 0.1% nucleotides in the diets with varying FM levels was able to partially prevent the development of marked histological alterations in the hepatopancreas of shrimp. No other remarkable differences were observed between study groups.

### 3.6. Challenge Test

**Figure 5:**
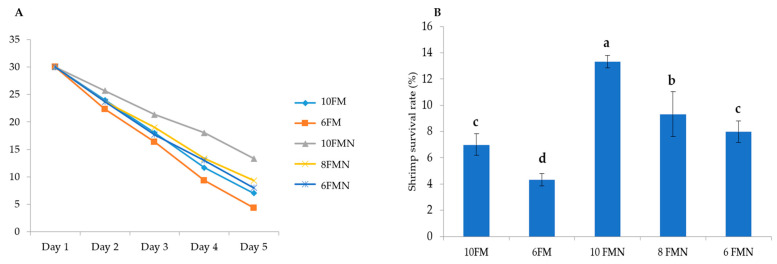
(**A**) Evolution of mean survival rate in Pacific white shrimp (PWS) over time in each study group after challenge test with *Vibrio harveyi* infection at dose of 1 × 10^5^ CFU mL^−1^, intramuscularly; (**B**) mean survival rate at the end of the challenge test. Different letters indicate statistically significant differences (*p* < 0.05) between the study groups. 10FM (control group; 10% FM), 6FM (6% FM and no nucleotide supplementation), 10FMN (10% FM; 0.1% nucleotides), 8FMN (8% FM; 0.1% nucleotides) and 6FMN (6% FM; 0.1% nucleotides).

Nucleotide supplementation enhanced disease resistance, as evidenced by a significantly higher survival rate upon *V. harveyi* challenge in all nucleotide-supplemented groups, compared to both 10FM and 6FM (Figure 5). Death occurred mostly from 18 to 32 h after infection, with PWS showing signs of weakness, passive swimming on the surface, muscle cramps, milky white abdominal muscles, and reddish-yellow coloration of the hepatopancreas.

### 3.7. Organoleptic Evaluation

The results of the overall scores obtained in each parameter including shrimp color, aroma, flavor, and texture are shown in Table 5. Nucleotides had no remarkable impact (neither positive nor negative). Numerically, the flavor of the shrimp in the 10FM and 10FMN groups was slightly sweeter than that of the rest of the dietary treatments, and these groups also showed a better texture compared to the others.

### 3.8. Profitability

The use of dietary nucleotides resulted in increased profitability. A 5.47% higher profit was achieved with 10FMN (gross margin = IDR 148,461,501), compared to 10FM (gross margin = IDR 140,757,291). A 10.95% higher profit was also obtained with 6FMN (gross margin = 140,010,741), compared to 6FM (126,197,614). On the other hand, similar profits were achieved with 6FMN and 10FM (gross margin = IDR 140,010,741 vs. IDR 140,757,291).

## 4. Discussion

Nucleotides are low-molecular-weight intracellular compounds which, besides being the building blocks of DNA and RNA, play important roles in physiological and biochemical processes [20,34,35]. They have also been reported to improve shrimp health by modulating their immune response [21,36,37]. The results from this study indicate that the use of nucleotides (Nucleoforce^®^) improves growth performance and profitability in PWS and, especially, allows greater disease resistance. More specifically, in PWS receiving diets in which FM has been partially replaced with vegetable protein sources, nucleotide supplementation led to better FBW, PWG, FCR, TGC, ADG, and higher economic profits, as well as a healthier hepatopancreas and increased survival rates upon *Vibrio harveyi* challenge. On the other hand, in PWS receiving a commercial diet with regular FM levels, nucleotide supplementation allowed higher disease resistance and no inferiority in terms of performance, immune response, and profitability, without altering the organoleptic parameters.

The main positive impact of nucleotide supplementation in PWS observed in this study is probably the improvement seen in growth performance. As the trend in reducing FM usage continues, this beneficial effect would support the incorporation of nucleotides in such circumstances during PWS production. This is in line with the study by Andrino et al. [18], where the inclusion of 0.2% nucleotides produced significantly better specific growth rates (SGR) and feed conversion efficiency (FCE) compared to shrimp without dietary nucleotides after a 60-day feeding trial. In our previous study, despite not reaching statistical significance, the supplementation of 0.1% nucleotides led to better growth, especially in the group in which FM had been partially replaced with SBM, compared to the control diet [21]. Results from the present study confirm the efficacy of nucleotides in improving PWS growth performance up to commercial size, complementing the positive outcomes of the prior study [21]. The role of nucleotides and metabolites in aquatic organisms has been studied now for a little over two decades [35], and the positive effects of various nucleotide products on growth and feed utilization performance of fish and crustaceans are well established [34,38,39,40]. However, there are still very few reports explaining how nucleotides work or how they enhance the growth performance of PWS. Metailler et al. [41] and Hossain et al. [34] argue that the feed attractant properties of nucleotides promote the rapid intake of the feed which prevents the leaching of nutrients allowing the shrimp to benefit, especially by supporting growth performance, from the complete nutritional properties contained in the diet. In this study, we observed the effects of prolonged nucleotide administration on the growth of PWS, up until they reached their consumption size. We noted that the 110-day administration period with intensive culturing in an open pond system provides a clear positive trend towards increased *L. vannamei* growth compared to the 70-day results obtained in our previous study [21].

Nucleotides modulate the immune response, and this could be seen with the improved results in the immune parameters evaluated during this trial, namely THC and lysozyme activity. Unlike in previous experiments [21], here we did not see a significant effect in this regard. Nevertheless, the trends indicate that there was probably an effect that might have been significant, if only restricted to the initial and more vulnerable stages of early shrimp development.

In crustaceans, circulating hemocytes play an important role in immunological responses [42,43]. However, environmental stressors, especially in open pond systems, could induce apoptosis of hemocytes in the shrimp [44], leading to decreases in THC, and hypothetically, this could induce deterioration of the immune system [44,45]. Prior studies by Murthy et al. [46] and Shankar et al. [47] showed that THC significantly increased with nucleotide supplementation. This indicates that the administration of nucleotides might stimulate the release and activation of hemocytes. Hemocytes also produce cytotoxic molecules, such as lysozymes, that are involved in the degradation of microbes within and outside the hemocytes [48]. The benefits of the activation of lysozyme activity were confirmed after feeding the kuruma shrimp, *Marsupenaeus japonicus*, with nucleotide-rich baker’s yeast [49].

Infection with *Vibrio harveyi* is an important issue to be considered in shrimp production. Data from this study demonstrate a remarkable positive effect of nucleotides, as all supplemented groups achieved higher survival rates. Adding nucleotides to a diet with 10% FM resulted in significantly higher resistance to the challenge compared to 6% FM, but also to 10% FM, without nucleotides. The use of nucleotides facilitated a higher survival rate even in a diet with 8% FM when compared to 10% FM (i.e., with higher FM, but without nucleotides). This is consistent with prior observations in shrimp also challenged with *V. harveyi* under different conditions [21]. It has been reported that with the ability of nucleotides to induce the activation of specific and nonspecific immune systems, dietary nucleotides can enhance the resistance of aquatic organisms against various pathogens, including viral, bacterial, and parasitic pathogens [34]. A previous study by Andrino et al. [18]. revealed that *L. vannamei* survival was higher in the group fed with nucleotides compared to those fed without dietary nucleotides. The significantly higher survival rate of shrimp fed with dietary nucleotides in our study is in line with our previous study where all groups fed with nucleotide-supplemented diets reached significantly higher survival rates [21]. The protective effects of nucleotides are probably due to an accelerated immune response to disease and environmental changes.

In the present study, the whole-body proximate composition indicated a higher protein deposition in the groups receiving dietary nucleotides compared to the group without dietary nucleotides. In addition, we found comparable values for other nutritional profiles, including total fat, moisture content, carbohydrate, and crude fiber content. The higher level of protein in the nucleotide-supplemented groups might be attributed to the ability of dietary nucleotides to influence protein biosynthesis by regulating the intracellular pool of nucleotides [34]. This is closely related to the role of nucleotides as the building blocks for DNA, which is further transcribed and translated to form protein in the shrimp body [34,50]. Numerically, the addition of nucleotides also increased the ash content within the whole body of the shrimp. This effect could be linked to the nature of the nucleotides used in this study, as a balanced concentrate of free nucleotides and active precursors, obtained from yeast. In a previous study, Tacon and Cooke [51] reported that the dietary inclusion of nucleotides in the form of yeast extract increased the ash content of rainbow trout carcass.

The main site for the absorption of nutrients in shrimp is the hepatopancreas [52]. This tubular system facilitates the rapid transport of cellular nutrients to the hemolymph, including dietary nucleotides. Thus, it is important to maintain the hepatopancreas in optimum condition. In the present study, the hepatopancreas of shrimp fed with 10% FM showed a normal structure without any cell structure damage. However, PWS fed with lower FM levels showed alterations such as hemocyte leaching and enlargement of the nucleus and vacuolation. Anti-nutritional factors in SBM might constitute an important factor responsible for the abnormal intestinal condition and reduced nutrient absorption [53]. A prior study by Yao et al. [54] indicated that the use of high SBM levels in feeds could induce intestinal damage and significantly decrease cell membrane thickness in the hepatopancreas of PWS. In the study herein, the inclusion of 0.1% nucleotides was capable of partially preventing the development of marked histological hepatopancreas alterations observed in the shrimp fed with 8% and 6% FM. The benefits of adding nucleotides to the diet may be due to their low molecular weight, which favors optimum digestion and absorption processes in the hepatopancreas.

Reports on the organoleptic evaluation of shrimp fed with functional or alternative ingredients are scarce. A study by Soller et al. [55] reported no statistical differences in the texture, appearance, aroma, or flavor between the shrimp fed diets containing menhaden fish oil and soybean oil. In our study, panelists were unable to distinguish shrimp from the different groups based on their color, aroma, flavor, or texture, which means that nucleotide supplementation does not alter these organoleptic parameters. It should also be highlighted that a better texture and sweeter flavor was achieved with nucleotides. Therefore, perhaps the use of nucleotides in diets might provide a positive influence on the sensory attributes of shrimp when FM is reduced.

Profitability is a key parameter in shrimp production, and feed-associated costs constitute a major variable in shrimp production from an economic standpoint. It is known, and can also be clearly appreciated in our study, that large-scale replacement of FM by vegetable protein leads to reduced profitability due, mainly, to impaired growth performance and health, despite savings in feed costs. That is why the use of high-quality alternative protein sources is preferred when replacing FM [56]. In addition, FM replacement strategies can also incorporate the use of complementary ingredients to obtain a more balanced nutrient profile and increase nutrient utilization [56,57]. Incorporating nucleotides does increase the cost, although not substantially, as the inclusion level is rather low. On the other hand, as mentioned previously, adding nucleotides leads to better disease resistance, which can enhance the benefits obtained in each shrimp production batch. As a result, in this study we managed to show that, overall, nucleotides supplementation generates an increased return on investment to the farmer. More specifically, adding nucleotides to PWS diets with reduced FM leads to a higher gross margin thanks to the markedly improved performance, and despite the slight increased investment required initially. Furthermore, profitability in 10FMN is quite similar to that of 10FM, but 10FMN provides better disease resistance.

Further studies are warranted in order to further validate the effects of nucleotides in this animal species as well as to support the approach suggesting their ability to counteract the negative effects of replacing FM by SBM in shrimp diets. This could be done in other countries with similar trends, like in Vietnam.

## 5. Conclusions

This study demonstrated that dietary nucleotide supplementation in PWS receiving diets in which FM has been partially replaced with vegetable sources leads to improved growth performance, better disease resistance and greater profitability. In addition, the inclusion of nucleotides did not affect the organoleptic features of shrimp.

Based on the positive results attained in this study, the 0.1% dietary nucleotide supplementation could be considered as a complementary tool to enhance shrimp production cultured under intensive open-pond systems.

## Figures and Tables

**Table 1:** Composition (%, *as is*) of diets used in the study. Diets were administered to Pacific white shrimp (PWS) for 110 days, and were formulated using different levels (10%, 8% and 6%) of fish meal (FM), and with or without adding dietary nucleotides (N).

Ingredient (%, *as Is*)	Diet				
	10FM	6FM	10FMN	8FMN	6FMN
Menhaden fish meal ^1^	10.00	6.00	10.00	8.00	6.00
Soybean meal ^1^	43.00	49.50	43.00	44.80	49.50
Corn gluten meal ^1^	10.00	10.00	10.00	10.00	10.00
Menhaden fish oil ^1^	5.64	5.64	5.64	5.64	5.64
Soy-Lecithin ^2^	1.00	1.00	1.00	1.00	1.00
Nucleotides ^3^	**0.00**	**0.00**	**0.10**	**0.10**	**0.10**
Corn starch ^1^	8.06	5.56	7.96	8.16	5.46
Wheat products ^4^	17.00	17.00	17.00	17.00	17.00
Mineral premix ^2^*	0.70	0.70	0.70	0.70	0.70
Vitamin premix ^2#^	1.90	1.90	1.90	1.90	1.90
KP-dibasic ^2^	2.50	2.50	2.50	2.50	2.50
Choline chloride ^2^	0.20	0.20	0.20	0.20	0.20
**Formulation cost (IDR/Kg)**	13.137	12.727	13.688	13.489	13.278
**Formulation cost (USD/Kg) ^5^**	0.9178	0.8892	0.9553	0.9414	0.9267

10FM (control group; 10% FM), 6FM (6% FM and no nucleotide supplementation), 10FMN (10% FM; 0.1% nucleotides), 8FMN (8% FM; 0.1% nucleotides) and 6FMN (6% FM; 0.1% nucleotides). ^1^ PT FKS Multi Agro, Tbk. Jakarta, Indonesia. ^2^ PT Fenanza Putra Perkasa, Jakarta, Indonesia. ^3^ Nucleoforce^®^, Bioiberica, S.A.U, Palafolls, Spain. ^4^ PT Pundi Kencana, Cilegon, Banten, Indonesia. ^5^ Currency conversion on 15 January 2022: USD 1 = IDR 14.310. * Mineral premix (g/100 g premix): 0.004 cobalt chloride, 0.550 cupric sulfate pentahydrate, 2.000 ferrous sulfate, 13.862 magnesium sulfate anhydrous, 0.650 manganese sulfate monohydrate, 0.067 potassium iodide, 0.010 sodium selenite, 3.193 zinc sulfate heptahydrate, and 69.664 alpha-cellulose. ^#^ Vitamin premix (g/kg premix): 4.95 thiamin·HCL, 3.83 riboflavin, 4.00 pyridoxine HCL, 10.00 Ca-pantothenate, 10.00 nicotinic acid, 0.50 biotin, 4.00 folic acid, 0.05 cyanocobalamin, 25.00 inositol, 0.32 vitamin A acetate (500,000 IU/g), 80.00 vitamin D3 (1,000,000 IU/g), 0.50 menadione, and 856.81 alpha-cellulose.

**Table 2:** Proximate analysis, calories, and amino acid (AA) composition (% *as is*, dry matter basis) of experimental diets utilized in this trial. Diets were administered to Pacific white shrimp (PWS) for 110 days, and were formulated using different levels (10%, 8% and 6%) of fish meal (FM), and with or without adding dietary nucleotides (N).

	Diet Code				
	10FM	6FM	10FMN	8FMN	6FMN
Proximate analysis (%, *as is*) *					
Protein content	36.55	36.07	36.95	36.57	35.99
Fat content	6.75	6.00	6.07	6.28	6.22
Moisture content	7.64	7.58	8.70	9.08	9.23
Crude fiber	2.69	3.40	1.75	1.91	1.63
Calories (kcal/100 g) *					
Calories from fat	60.75	54.00	54.63	56.52	64.98
Total calories	362.47	350.32	343.67	345.32	350.82
Amino acid profile (%, *as is*) *					
L-Serine	1.92	2.09	2.28	2.18	1.97
L-Glutamic acid	6.87	6.16	4.96	5.49	5.89
L-Phenylalanine	3.32	2.75	3.78	3.27	2.43
L-Isoleusine	1.26	1.41	1.41	1.39	1.39
L-Valine	1.37	1.53	1.56	1.51	1.50
L-Alanine	1.63	1.68	1.61	1.61	1.69
L-Arginine	2.48	2.88	3.49	3.21	2.63
Glycine	1.73	1.81	2.13	1.93	1.74
L-Lysine	2.86	1.71	1.60	1.80	2.23
L-Aspartic acid	3.00	3.25	2.52	2.79	3.27
L-Leusine	2.65	2.88	2.94	2.90	2.81
L-Tyrosine	1.36	1.63	2.30	2.05	1.45
L-Proline	2.02	2.05	2.00	2.01	2.01
L-Threonine	1.65	1.81	2.09	1.93	1.71
L-Histidine	1.05	1.22	1.62	1.43	1.12
L-Cystine	0.35	0.52	0.48	0.44	0.58
L-Methionine	0.65	0.67	0.68	0.68	0.68
L-Tryptophan	0.32	0.36	0.33	0.34	0.33

10FM (control group; 10% FM), 6FM (6% FM and no nucleotide supplementation), 10FMN (10% FM; 0.1% nucleotides), 8FMN (8% FM; 0.1% nucleotides) and 6FMN (6% FM; 0.1% nucleotides). * Analyses conducted by the Saraswanti Indo Genetech Laboratory, Bogor, West Java, Indonesia.

**Table 3:** Growth performance of Pacific white shrimp (PWS) fed diets supplemented with nucleotides (N) and different levels of replacement of fish meal (FM) by vegetable protein sources for 110 days. Values represent the mean of ten replicates. Results in the same columns with different superscript letter are significantly different (*p* < 0.05).

Diet	Final Biomass (g)	FBW (g)	Survival Pre-Challenge (%)	PWG (%)	FCR	TGC	ADG
10FM	7604.0	20.05 ^a^	83.31	1904.66 ^a^	1.36 ^a^	0.05429 ^a^	0.173 ^a^
6FM	7405.5	19.44 ^b^	83.91	1844.15 ^b^	1.40 ^b^	0.05341 ^b^	0.167 ^b^
10FMN	7652.5	20.28 ^a^	83.84	1928.38 ^a^	1.34 ^a^	0.05462 ^a^	0.175 ^a^
8FMN	7521.0	19.98 ^a^	84.67	1897.90 ^a^	1.37 ^a^	0.05419 ^a^	0.173 ^a^
6FMN	7534.0	19.95 ^a^	83.93	1895.46 ^a^	1.37 ^a^	0.05415 ^a^	0.172 ^a^
*p*-value	0.1827	<0.0001	0.9336	<0.0001	<0.0001	<0.0001	<0.0001
PSE	116.0961	0.1449	1.3703	14.4975	0.0106	0.0002	0.0013

10FM (control group; 10% FM), 6FM (6% FM and no nucleotide supplementation), 10FMN (10% FM; 0.1% nucleotides), 8FMN (8% FM; 0.1% nucleotides) and 6FMN (6% FM; 0.1% nucleotides), FBW (final body weight), PWG (percentage weight gain), FCR (feed conversion ratio), TGC (thermal growth coefficient), ADG (average daily growth in gram per day), PSE (pooled standard error).

**Table 4:** Proximate composition (%, *as is*), calories from fat and total calories of feed (KCal 100 g^−1^), and amino acid profile (%, *as is*) of Pacific white shrimp (PWS) whole body at the end of the 110-day growth trial.

Parameter	Unit	Nutritional Composition					
		Control	10FM	6FM	10FMN	8FMN	6FMN
Protein content	%	24.89	22.42	22.29	24.72	24.88	23.65
Total fat	%	1.01	0.28	0.35	0.31	0.33	0.29
Moisture content	%	79.69	68.72	72.77	69.43	69.96	69.72
Carbohydrate	%	0.65	1.56	2.45	1.88	1.70	2.14
Ash content	%	3.76	1.59	1.78	1.71	2.27	1.82
Calories from fat	KCal 100 g^−1^	4.09	4.41	4.38	4.20	4.41	4.58
Total calories	KCal 100 g^−1^	71.25	113.53	109.34	111.45	115.77	110.66
Crude fiber	%	0.44	<0.02	<0.02	<0.02	<0.02	<0.02
L-Cystine	%	0.23	6.44	5.31	6.02	6.14	5.89
L-Methionine	%	0.09	4.55	4.38	4.47	4.78	4.66
L-Serine	%	0.37	11.53	11.02	11.15	11.53	11.44
L-Glutamic acid	%	1.39	31.36	29.35	32.52	32.25	31.89
L-Phenylalanine	%	0.71	15.25	13.74	15.29	15.44	15.12
L-Isoleucine	%	0.39	11.15	10.95	11.44	10.87	10.99
L-Valine	%	0.45	10.77	10.82	11.34	11.33	10.39
L-Alanine	%	0.74	12.63	12.68	12.65	12.72	12.77
L-Arginine	%	0.40	28.44	23.11	28.65	28.23	26.44
Glycine	%	1.10	17.13	17.74	17.63	19.13	18.66
L-Lysine	%	0.53	14.56	14.22	15.07	14.37	14.44
L-Aspartic acid	%	0.75	16.89	17.02	18.45	17.23	16.89
L-Leucine	%	0.71	18.66	17.74	16.55	17.75	17.66
L-Tyrosine	%	0.52	11.41	10.39	10.45	10.48	10.59
L-Proline	%	0.41	18.78	18.85	19.02	19.11	18.59
L-Threonine	%	0.58	11.30	10.54	11.33	11.40	10.77
L-Histidine	%	0.40	7.77	7.42	7.51	7.96	7.88
L-Tryptophan	%	0.11	1.65	1.86	1.91	1.79	1.82

10FM (control group; 10% FM), 6FM (6% FM and no nucleotide supplementation), 10FMN (10% FM; 0.1% nucleotides), 8FMN (8% FM; 0.1% nucleotides) and 6FMN (6% FM; 0.1% nucleotides).

**Table 5:** Results of organoleptic assessment of Pacific white shrimp (PWS) fed with different diets for 110 days (n = 15 per group).

Treatment	Observed Parameter			
	Color	Aroma	Flavor	Texture
10FM	3.45	3.45	3.60	3.66
6FM	3.35	3.35	3.46	3.35
10FMN	3.65	3.55	3.66	3.65
8FMN	3.40	3.46	3.55	3.55
6FMN	3.45	3.66	3.55	3.35
*p*-value	>0.05	>0.05	>0.05	>0.05

10FM (control group; 10% FM), 6FM (6% FM and no nucleotide supplementation), 10FMN (10% FM; 0.1% nucleotides), 8FMN (8% FM; 0.1% nucleotides) and 6FMN (6% FM; 0.1% nucleotides).

## Data Availability

The data presented in this study are available on request from the corresponding author.
